# Experimental and Theoretical Electron Paramagnetic Resonance (EPR) Study on the Temperature-Dependent Structural Changes of Methylsulfanylmethane

**DOI:** 10.3390/ijms12084909

**Published:** 2011-08-03

**Authors:** Recep Tapramaz, Ercan Türkkan, Ömer Dereli

**Affiliations:** 1 Ondokuz Mayis University, Samsun 55100, Turkey; 2 Selcuk University, Konya 42030, Turkey; E-Mails: eturkkan@selcuk.edu.tr (E.T.); odereli@selcuk.edu.tr (Ö.D.)

**Keywords:** EPR, methylsulfanylmethane, radiation damage, radical

## Abstract

Methylsulfonylmethane (or dimethyl sulfone), a naturally produced and vitally important organosulfur compound in living organisms, was irradiated with gamma rays, and the produced radicals were investigated using electron paramagnetic resonance spectroscopy at different temperatures. The structure and behavior of the radical changed when the temperatures varied. The hyperfine splitting of the CH_3_ group was small, and the ^33^S splitting was relatively high between 80 and −50 °C. When the temperature was between −50 and −160 °C, the ^33^S splitting became small and the CH_3_ splitting was higher. However, the group kept rotating; therefore, only the isotropic splitting values were measured, and the *g*-values were anisotropic. When the temperature decreased below −180 °C, the CH_3_ group stopped rotating, and the hydrogen splitting values became nonequivalent due to an inhomogeneous electron distribution. The observed structures can be explained by referring to both the experimental and theoretically calculated values reported.

## 1. Introduction

The organic compound (CH_3_)_2_SO_2_ has several names, e.g., dimethyl sulfone (DMSO_2_), methylsulfanylmethane (MSM) or methyl sulfone, and it is an important organic compound in chemistry, especially in the health sciences. MSM is structurally similar to its metabolite dimethyl sulfoxide (DMSO), which is a well-known polar organic solvent, except that MSM has one more oxygen atom and a higher melting point. Although some properties are similar to DMSO, MSM has some unusual properties for an organic compound, for example it is found in a white crystalline form at room temperature and it melts at 109 °C. The oxidation number of MSM is higher, which indicates more reactivity in biological media relative to DMSO having lower oxidation number. In addition, MSM is odorless, tasteless, water-soluble, aprotic and is a highly polar molecule. Molten MSM is used as a solvent for a wide variety of compounds, including organics, polymers and inorganic salts.

MSM and DMSO are naturally occurring organosulfur compounds found in living organisms. They can be detected in human and animal fluids and tissues as well as in most common foods, such as milk, meat, fish, a variety of fruits, vegetables and grains [1–3]. However, when foods are processed with some chemicals, heated or dried, a significant amount of MSM is lost. Studies have indicated that maintaining a minimum concentration of MSM in the body may be critical to a normal functioning metabolism and the concentration level of MSM in the body decreases with increasing age [4–6]; therefore, if a person’s diet consists primarily of raw foods, it is likely that the person is receiving enough MSM for proper health. MSM has an extremely low toxicity and is extensively used in cosmetics and pharmaceutics [7,8]. In addition, MSM has high radical scavenging capabilities [9] that are similar to DMSO, and its radical scavenging properties have been studied by many groups [10–13].

EPR spectroscopic study was carried out by Andersen on x- irradiated MSM single crystals at 77 K (−196 °C) [14]. A freely rotating ĊH_3_ radical was observed below −120 °C. When the temperature was increased, the ĊH_3_ radical diminished irreversibly and some new species appeared. The central intense and stable singlet, and the weak ^33^S HFCC lines reported at higher temperatures look similar to the radical being discussed in this paper, but there is no further information about the radical when the sample is cooled down again.

Another work on MSM was made by Kasai on condensed MSM and Na atoms in frozen argon matrix at 77 K and were UV irradiated using Xe-Hg lamp [15]. A freely rotating ĊH_3_ radical spectrum was clearly observed.

An electron paramagnetic resonance (EPR) spectroscopic study of gamma-irradiated DMSO was also carried out at low temperatures by Nishikida and Williams because DMSO melts at approximately 18 °C, and the (CH_3_)SO^−^ ionic radical was observed. However, the behavior of the radical could not be studied at higher temperatures because of lower melting point [16]. The structural differences will be discussed below.

EPR spectroscopy is one of the most powerful methods used for studying the structure and determining the identity of paramagnetic centers or radicals via the *g*–value and the hyperfine coupling coefficient, [HFCC hereafter], because the nuclei are near the unpaired electron. The HFCC of a given nucleus in a radical is highly sensitive to its chemical environment. Hence, HFCC can be used to determine the spin-density distribution of the radical and also to deduce valuable information about the identity and structure of the radical. However, the *g*-value depends on the spin distribution throughout the radical; thus, it can be significantly affected by intermolecular interactions. Extraction of this information from experimental spectra is not always straightforward, and therefore, quantum-chemical calculations are frequently needed [17–21].

In this study, the structural and magnetic properties of the radicals induced in gamma-irradiated MSM at room-temperature [(CH_3_)_2_SO_2_] single crystals were investigated using EPR spectroscopic technique between +90 °C, which is just below the melting point, and −185 °C. The theoretical calculations were used to support the interpretation of the experimental results and to assist in the identification of the radical type by comparing the experimental spectra. The structure and the behavior of the radicals formed in MSM single crystals after gamma irradiation at room temperature seem to be different and the behaviors at low temperatures are explained.

### 1.1. Theoretical Considerations

The hyperfine splitting parameters describe the interactions between unpaired electrons and magnetic nuclei in a paramagnetic center. The 3 × 3 dimensional hyperfine tensor **A** can be separated into its isotropic and anisotropic (dipolar) components. The isotropic hyperfine splitting *A_iso_* (*N*) caused by nucleus *N* is equal to the Fermi contact term and is related to spin densities *ρ^α^^−^^β^* (*R_N_*) on the corresponding nuclei by the following equation:

(1) $$A_{\mathrm{iso}}(N)=\frac{4\pi }{3}\beta_{e}\;\beta_{N}\;g_{e}\;g_{N}\;{\langle S_{Z}\rangle }^{-1}\rho_{N}^{\alpha -\beta }$$

where *β_e_* and *β_N_* are the electronic and nuclear Bohr magnetons, respectively, *g_e_* and *g_N_* are the free-electron and nuclear *g*-values, respectively, 〈*S_Z_*〉 is the expectation value of the *z*-component of the total electronic spin, and $\rho_{N}^{\alpha -\beta }$ is the spin density on the nucleus *N* at the position *R_N_*. The anisotropic hyperfine tensor components *T_kl_* in the Cartesian coordinate axes *k* and *l* in the first-order approximation are given by the following equation:

(2) $$T_{\mathrm{kl}}\;(N)=\frac{1}{2}\beta_{e}\;\beta_{N}\;g_{e}\;g_{N}\;{\langle S_{Z}\rangle }^{-1}\sum_{\mu ,\nu }P_{\mu ,\nu }^{\alpha -\beta }\langle \varphi_{\mu }\left|r_{N}^{-5}(r_{N}^{2}\;\delta_{\mathrm{kl}}-3r_{N,k}\;r_{N,l})\right|\varphi_{\nu }\rangle$$

where **r***_N_* *=* **r** − **R***_N_* and $P_{\mu ,\nu }^{\alpha -\beta }$

represent the spin-density matrix elements. Indices *k* and *l* cyclically represent the *x*, *y* and *z*-axes. The ***T*** tensor is always traceless and may be brought into diagonal form with diagonal elements *T_xx_*, *T_yy_* and *T_zz_*, where *T_xx_* + *T_yy_*+*T_zz_* = 0. The isotropic and anisotropic (dipolar) components contain information about the spin densities of the unpaired electron on the nuclei in the neighborhood [22].

The ***g***-tensor components *g_rs_* are calculated using the coupled-perturbed density functional theoretical (CP-DFT) formulation of Neese and Estebes *et al* involving four terms [23,24]:

(3) $$g_{\mathrm{rs}}=g_{e}\delta_{\mathrm{rs}}+\Delta g^{\mathrm{RMC}}\delta_{\mathrm{rs}}+\Delta g_{\mathrm{rs}}^{\mathrm{GC}}+\Delta g_{\mathrm{rs}}^{\mathrm{OZ}/\mathrm{SOC}}$$

The first term is the isotropic contribution representing the free-electron *g*-value. The second term of Equation 3 is a relativistic mass-correction term introduced by Angstl [25] to correct *g*-values for planar-aromatic complexes, which is calculated with the ground state spin-density and kinetic energy integrals as follows:

(4) $$\Delta g^{\mathrm{RMC}}=-\frac{\alpha^{2}}{S}\sum_{\mu ,\nu }P_{\mu \nu }^{\alpha -\beta }\langle \varphi_{\mu }|\hat{T}|\varphi_{\nu }\rangle$$

where *α* is the fine-structure constant, *S* is the total spin of ground state, $P_{\mu \nu }^{\alpha -\beta }$

is the spin-density matrix, *φ* is the basis set, and *T̂* is the kinetic-energy operator. The third term of Equation 3 is a diamagnetic correction introduced by Stone [26] and is calculated depending on the ground-state spin density as follows:

(5) $$\Delta g^{\mathrm{GC}}=\frac{1}{2S}\sum_{\mu ,\nu }P_{\mu \nu }^{\alpha -\beta }\langle \varphi_{\mu }|\sum_{N}\left[\frac{\alpha^{2}Z_{\mathrm{eff}}^{4}}{2{\left|r_{i}-R_{N}\right|}^{3}}\right](r_{N}\;r_{O}-r_{N,s}\;r_{O,s})|\;\varphi_{\nu }\rangle$$

where ***r****_N_* is the position vector of the electron relative to the nucleus *N*, ***r****_O_* is the position vector relative to gauge origin, *Z_eff_* is the effective nuclear charge, and the term in square brackets is the effective spin–orbit coupling of the *i*^th^ electron at nucleus *N*. The fourth term of Equation 3 is the dominant correction and comes from crossed terms between the Zeeman orbital (OZ) operator and the spin–orbit coupling. This term is calculated using Neese’s coupled perturbed theory and the DFT methodology (CP-DFT) [23].

### 1.2. Computational Details

**Figure 1.**
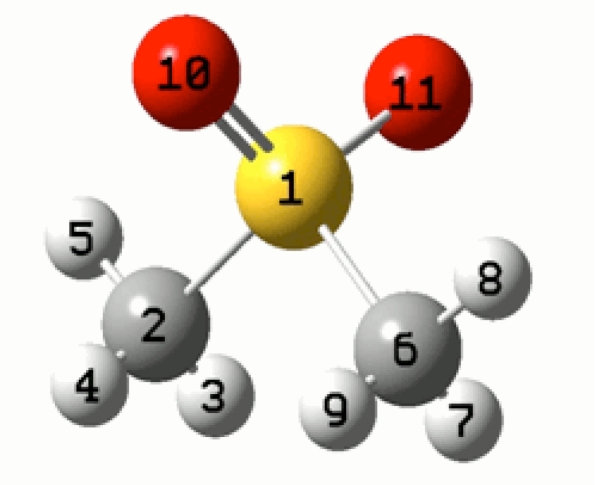
Methylsulfanomethane (MSM) [(CH_3_)_2_SO_2_] molecule. The Ball 1 is S, balls 10 and 11 are ^16^O, and the balls 2 and 6 are ^12^C and the remaining balls are ^1^H.

From the assumptions made on the experimental spectra, the possible radicals that formed after gamma irradiation were modeled using theoretical computations. The molecular geometry parameters of MSM [(CH_3_)_2_SO_2_] that are shown schematically in Figure 1 were taken from crystal data [27–29]. The geometry optimizations of the model radicals were made with the UMP2 method and the standard 6-311++G(d,p) basis set. The optimizations were performed without any constraints (full optimization). All stationary points were confirmed as local minima by their harmonic vibration frequencies, and the normal-mode calculations were performed at the same level as the geometry optimizations.

The hyperfine and *g-*values of the modeled radicals were calculated by the UB3LYP method using the TZVP basis set [30] because a successful prediction of the EPR parameters for the sulfur containing radicals has been recently demonstrated using this method/basis set combination by Hermosilla *et al.* [31]. All geometry optimizations and EPR parameter calculations were performed using the GAUSSIAN 03 program [32].

## 2. Results and Discussion

For simplicity, the results and discussion have been separated by the EPR spectra of gamma-irradiated MSM at room temperature and at different temperatures, *i.e.*, at room temperature, at higher temperatures close to the melting point and at low temperatures.

### 2.1. Room Temperature Spectra

**Figure 2.**
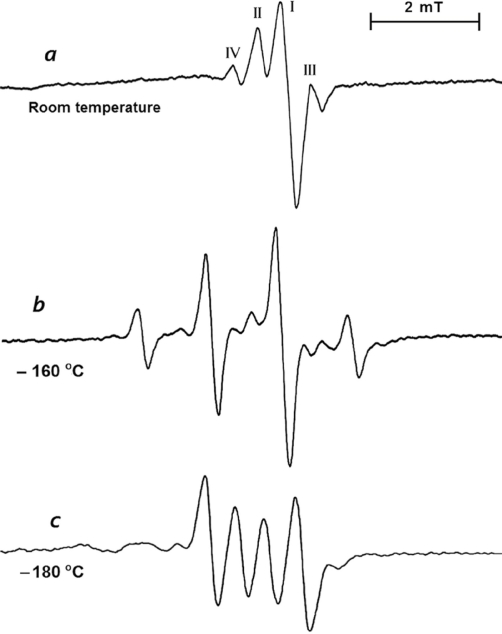
Electron paramagnetic resonance (EPR) spectra of gamma irradiated methylsulfanylmethane (MSM) single crystal at (**a**) room temperature; (**b**) −160 °C; and (**c**) −180 °C along *b* axis.

**Figure 3.**
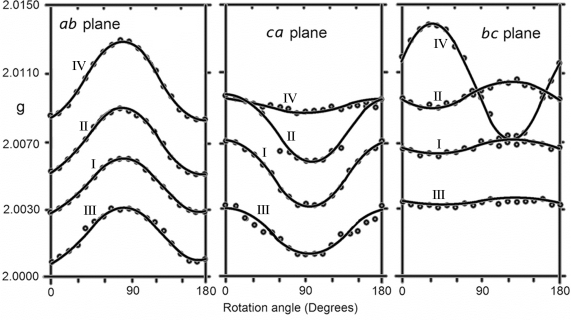
The plot of the positions of all lines observed in MSM single crystal at room temperature in mutually perpendicular planes.

Figure 2a shows the EPR spectra when the crystalline *b* axis was parallel to the magnetic field. Four lines with labels I, II, III and IV were detected, and their angular variations in three perpendicular crystalline planes are shown in Figure 3. The angular variations of lines I, II and III are the same where the separation of lines II and III is fixed with the value of 1.2 mT and an axially symmetric *g*-value averaging 2.0062, which indicated that they belonged to the same radical. However, the intensity of line I was too high and was not comparable to the intensities of lines II and III; therefore, the separation did not arise from hyperfine interactions. Moreover, the width of line I changed slightly with orientation, which indicated some small and irresolvable HFCC values. Lines II and III may correspond to satellites arising from a small HFCC values contained in line I [18] and can be attributed to a sulfur-centered radical. Conversely, the intensity of line IV was too small compared to other lines and behaved differently with an average *g*-value of 2.0010. This line was completely quenched when heated up to 70 °C or after keeping it at room temperature for approximately two months. This line can be attributed to a ${\text{SO}}_{2}^{-}$

type ionic radical formed as an impurity after irradiation [33,34].

**Figure 4.**
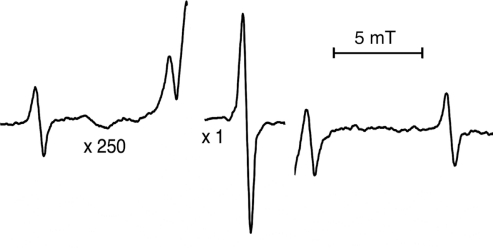
The hyperfine splitting lines due to ^33^S in gamma irradiated MSM single crystal at room temperature.

The ^33^S isotope has a nuclear spin of *I* = 3/2 and a natural abundance of 0.75%; therefore, when the spectrometer gain was increased by 200 times or more, four anisotropic lines caused by the ^33^S HFCC were clearly seen (Figure 4), and their *g*-value variation was exactly the same as those of line I in Figure 2a. The parameters measured at room temperature and evaluated with a second-order shift [18,21] are given in Table 1. The ^33^S HFCC was axially symmetric with an average value of 7.2 mT, which was greater than the value measured for the radical in DMSO around −50 °C [16] and was determined using *ab initio* molecular orbital calculations [35]. The unpaired electron occupied the 3s and 3p_z_ orbitals of the sulfur atom with ratios of 0.06 and 0.53, respectively.

### 2.2. High Temperature Spectra

When the temperature was increased from room temperature to +80 °C, the width of line I became narrower, and its amplitude increased due to the tumbling motion of the radical creating that line. Lines II and III showed no appreciable change, but line IV was irreversibly quenched with increasing temperature. When the temperature was increased above +80 °C, the quenching of all lines began because the melting point of MSM (+109 °C) was approached. The EPR parameters did not change significantly compared to the room temperature values.

### 2.3. Low Temperature Spectra

As the temperature decreased below −50 °C, line I in Figure 2a became weaker, and the ^33^S HFCC lines in Figure 4 started to quench; in addition, a new set of lines with an intensity distribution of 1:3:3:1 emerged and reached its highest point at −160 °C (Figure 2b). The HFCC was completely isotropic (1.3 mT), and the g-value was rhombic with average value of 2.0115 (Table 1). Similar spectra were seen in the gamma-irradiated DMSO at low temperatures and were attributed to CH_3_SO^−^ ionic radical by Nishikida and Williams [16] and Swarts *et al.* [35]. The HFCC values for the hydrogen atoms of the bound CH_3_ group were supposed to be equivalent, and the group kept rotating even at −160 °C. No ^33^S HFCC lines could be detected at low temperatures. In addition, the calculations showed that ^33^S lines became smaller and were under the intense spectra, Figure 2b.

When the temperatures decreased to −180 °C and below, the spectra changed further and showed four equally intense lines, Figure 2c. The angular variations of the lines showed that the spectra were created by two nonequivalent methyl protons because there were no other atoms in the structure other than hydrogen that could induce such spectra. The HFCC and *g*-values were axially symmetric and are given in Table 1. Both values differed from the values measured for the bound CH_3_ group at −160 °C.

**Figure 5.**
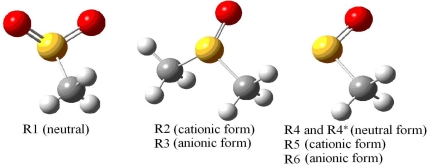
Optimized geometries of model radicals R1, R2, R3, R4, R4^*^, R5 and R6.

To assign the observed EPR parameters and determine the possible radical structure, a series of theoretical calculations were performed for several possible model radicals R1, R2, R3, R4, R5 and R6, Figure 5. The molecular structure of MSM obtained from the x-ray crystal data, Figure 1, was taken as the initial geometry. Model R1 was formed by removing the CH_3_ fragment from the molecule. Models R2 and R3 were cationic and anionic model radicals formed by removing an oxygen atom from the molecule. Model R4 was formed by removing one of the oxygen atoms and the CH_3_ fragment from the molecule. Models R5 and R6 were formed by cationic and anionic form of R4, respectively.

To provide accurate calculations for the hyperfine splitting and *g-*values, precise geometric structures of possible radicals were needed, and therefore, UMP2/6-311+G(d,p) level geometry optimizations were performed for six modeled radicals; the optimized radical geometries were then used as initial values to find the HFCC and *g*-values using the UB3LYP/TZVP level density-functional calculations. The theoretically calculated isotropic values of the hyperfine splitting and *g*-values of six model radicals are given in Table 2 with respect to the atomic numbering scheme shown in Figure 1.

In the calculations, the single and isolated molecule approach was used to perform DFT calculations assuming the radical was in gas phase at 0 K that is the lattice around the radical was not incorporated during EPR parameter calculations or geometry optimization calculations. The usefulness and feasibility of this methodology in the calculations of EPR spectroscopic parameters have previously been extensively demonstrated [22,36]. Moreover, 20% deviations, discussed below, in experimental and theoretical calculations include intrinsically the errors originating from environmental effects.

For most purposes of interpretation and assignment, 20% deviations would be quite acceptable for calculated isotropic hyperfine splitting values of experimentally isolated radicals [36]; the deviations in the calculated ^33^S HFCC values from the experimental spectra at room temperature were less than 20% for the R1 model radical. Furthermore, it was difficult to measure the isotropic *g*-values more accurately than 10^−3^, and for most applications, deviations up to 1000 ppm are considered to be satisfactory [23]. As seen in Table 2, the calculated isotropic *g*-value deviation of model radical R1 was approximately 100 ppm, which was closer to the room temperature experimental value when compared to other five model radicals. Therefore, it was reasonable to conclude that the theoretically calculated isotropic *g*-value of the radical and hyperfine splitting value of ^33^S for model radical R1 were in good agreement with experimental values at room temperature. Referring to the discussion on the intense line I in Figure 2a, the HFCC caused by the CH_3_ protons were less than 0.5 mT and were under the intense line. The CH_3_ groups that exist in radical species at higher temperatures freely rotate, and the three protons are assumed to be equivalent [37]. As seen in Table 2, the average HFCC of the three CH_3_ protons for the optimized model radical R1 was found to be 0.49 mT and was compatible with the observed radical at room temperature. This structure was not observed by Nishikida and Williams [16] because of the lower melting point of the host DMSO. Meanwhile the principal ^33^S HFCC value measured in this work (averaging 7.2 mT) is almost equal to the HFCC measured in x-irradiated MSM by Andersen and is appreciably higher than those of measured in DMSO (averaging 5.9 mT) by Nishikida and Williams [16]. We assumed that this difference originates from that the radical in DMSO contains only one oxygen atom while the radical in MSM contains two oxygen atoms and the spin population is relatively higher on S atom due to polarization effect of oxygen atoms.

As the temperature decreased between −50 °C and −160 °C, the electronic distribution and the structure of the radical changed as discussed above. The parameters obtained after the calculations showed that the model radical R4 fit the observed radical in Figure 5. The average HFCC and *g*-values for model radical R4 given in Table 2 were 1.08 mT and 2.012, respectively, and the difference in the experimental values (Table 1) was 500 ppm. The radical observed at low temperatures for the gamma-irradiated DMSO provided similar results [16] but no information was found for x-irradiated MSM at 77 K [14]. Taking the ^33^S splitting values at room temperature and CH_3_ proton splitting values for radicals in DMSO and MSM between −50 down to −160 °C, and also the temperature dependent anti-symmetric stretching mode and hence the elongation of S=O bonds (∼0.1 Å) of MSM molecule [38,39] we assumed that one of the oxygen atoms stayed closer to the S1 atom and the other one moved away in model radical R1; therefore, the radical behaved like model radical R4. The radical is basically a π electron radical, and the unpaired electron may have occupied the 3p_z_ orbital but not 3s orbital of the sulfur atom [35].

At temperatures of −180 °C and lower, the CH_3_ group stops rotating, and the hydrogen atoms become magnetically non-identical. One of the hydrogen atoms displayed an almost zero HFCC value, and the other two hydrogen atoms provided different and axially symmetric HFCC values. The *g*-values were also different from the radical observed at higher temperatures (Tables 1 and 2). The calculations showed that when the CH_3_ group stopped rotating and due to the polarization effect of lone pair electrons of the oxygen atom staying closer to sulfur atom, the unpaired electron density on the CH_3_ group shifted towards the two hydrogen atoms; as a result, the observed spectra were produced. According to the calculations which took care of the discussions made above for temperature interval of −50 down to −160 °C, one of the oxygen atoms stayed closer to the S1 atom and the other one went more away in the model radical R1, and therefore, the radical behaved like the model radical R4* below −180 °C. The calculated values (UB3LYP/TZVP) for the R4* structure, which was the modified model radical from R4, are given in Table 2 together with the model radical R4; it was only in this case that the HFCC values of two hydrogen atoms fit the experimental values. In another words, room temperature measurements and calculations indicate two oxygen atoms bound to sulfur atom [14–16] and low temperature measurements and calculations indicate only one oxygen atom. Moreover, S=O bonds are strong and cannot be broken easily. Therefore it seemed the only assumption that could be made to explain behaviors discussed above. The measured and calculated average values of the radical were all in the same order of magnitude and fit the acceptable limits [23,36].

## 3. Experimental Section

Crystalline MSM was purchased from Merck. Suitably sized single crystals were chosen and irradiated at room temperature with a ^60^Co γ–ray source at 0.818 kGy/h for approximately 36 h. The EPR spectra of an irradiated single crystal were recorded at +90, +20, −160 and −180 °C at three perpendicular crystalline planes with 10° intervals using a Varian E-109 Century Series X-band EPR spectrometer equipped with a Varian temperature control unit. Bruker’s Simfonia software was used for spectral simulations. The *g*-value corrections were made using a dpph sample (*g* = 2.0036).

The crystal structure of MSM was determined by x-ray diffraction analysis. The unit-cell structure was orthorhombic and can be classified by the following space groups: A_mma_ (D_2_*_h_*), A_21ma_ (C_1_*_v_*) or A_ma2_ (C_2_*_v_*). The unit cell dimensions were *a* = *b* = 7.36 Å and *c* = 8.00 Å, and the unit cell contained four molecules [27].

## 4. Conclusions

Gamma irradiated single crystals of (CH_3_)_2_SO_2_ (methylsulfanylmethane, MSM, or dimethyl sulfone) was investigated using EPR spectroscopy at different temperatures. Around room temperatures an intense line with average g value of 2.0062 and a doublet with the same g value and with constant splitting of 1.2 mT was observed which were assumed to be the weak satellites due to small hyperfine splitting. A weak line with average g value of 2.0010, which behaved differently and diminished in several months at room temperature, was also observed. This last line was attributed to ${\text{SO}}_{2}^{-}$type impurity. As the spectrometer gain was increased ^33^S hyperfine lines with the same g value and average hyperfine splitting value of 7.2 mT were observed. Molecular orbital calculations on several possible model radical structures showed that the radical was (CH_3_)ṠO_2_ which formed by loosing one of the CH_3_ groups. No proton splitting was observed.

- At higher temperatures near to melting point (109 °C) the line widths became narrower and lines became sharper but the spectra did not change appreciably.
- When the temperature was decreased between −50 °C and −160 °C, the central intense line split into 1:3:3:1 pattern with constant value of 1.3 mT. The average g value was measured to be 2.0115 which showed that the CH_3_ group keeps rotating, and ^33^S lines became smaller and lay under the intense lines. Calculations showed that the unpaired electron population shifted toward the CH_3_ group.
- When the temperature was decreased below 180 °C, the spectra converted to anisotropic two different 1:1 patterns with average *g* value of 2.0058, and average hyperfine values of 1.6 mT and 0.43 mT which showed that the CH_3_ group stopped rotation. Estimations on the structure with the help of molecular orbital calculations showed that one of the oxygen atoms of the radical gets closer to CH_3_ group and the other one goes away. The closer oxygen atom polarizes the unpaired electron distribution on the CH_3_ group and as a result the distribution on one of the hydrogen atoms becomes too small and on the other two oxygen atoms it becomes unequal.

## Supporting information

Optimized geometries in the Cartesian coordinates for R1, R2, R3, R4, R5, and R6 model radicals and the equilibrium geometry of the R4* model radical are shown in Table Q. This material is available via the Internet address: http://www.egitim.selcuk.edu.tr/izik/eturkkan/tableq.htm.

## Figures and Tables

**Table 1.** Measured EPR parameters of MSM single crystal at room temperature and +80 °C, −160 °C and −180 °C (Measurement errors: magnetic field ± 0.05 mT; *g* values ±0.0003).

**Room temperature spectra**				
Measured g values of line I, II–III doublet and ^33^S lines			Comment	
*g*_//_ = 2.0036	*g*_⊥_ = 2.0075	< *g* >=2.0062	II–III doublet splitting is 1.2 mT (constant)	
Measured g values of line IV				
*g*_//_ = 2.0143	*g*_⊥_ = 2.0078	< *g* >=2.0010		
Hyperfine coupling constants of ^33^S lines (mT).				
*A*_//_ = 9.1	*A*_⊥_ = 6.2	< *A* >= 7.2	*g* values are the same as those of line I.	
**Spectra at −160 °C**				
*g_x_* = 2.0076	*g_y_* = 2.0109	*g_z_* = 2.0160	< *g* >=2.0115	Hyperfine coupling constant of three equivalent methyl protons are isotropic with the value of 1.3 mT. ^33^S hyperfine lines could not be detected.
**Spectra below −180 °C**				
*A*_//_ = 2.6	*A*_⊥_ = 1.1	< *A* >=1.6	For one of the CH_3_ hydrogen atoms	
*A*_//_ = 0.3	*A*_⊥_ = 0.5	< *A* >=0.43	For other hydrogen atom	
*g*_//_ = 2.0037	*g*_⊥_ = 2.0069	< *g* >= 2.0058	HFCC of third hydrogen atom is too small to measure. ^33^S hyperfine lines could not be detected.	

**Table 2.** Calculated (UB3LYP/TZVP) values of isotropic hyperfine values (mT) and *g*-values for R1, R2, R3, R4, R4*, R5 and R6 model radicals. R4* is the modified model radical from R4 where the CH_3_ group stops rotating below −180 °C.

**R1**		**R2**		**R3**	
**Atom**	***A_iso_***	**Atom**	***A_iso_***	**Atom**	***A_iso_***
**S1**	6.37	**S1**	4.33	**S1**	0.51
**H7**	−0.33	**H3**	2.17	**H3**	0.47
**H8**	−0.33	**H4**	−0.14	**H4**	−0.31
**H9**	0.82	**H5**	0.03	**H5**	0.28
		**H7**	2.17	**H7**	0.47
		**H8**	0.03	**H8**	0.28
		**H9**	−0.17	**H9**	−0.31
< *A*(*CH*_3_) >	0.49		0.79		0.35
< *g* >	2.0063		2.0089		2.0047
R4 and R4* (in parenthesis)		**R5**		**R6**	
**Atom**	***A_iso_***	**Atom**	***A_iso_***	**Atom**	***A_iso_***
**S1**	0.87 (0.80)	**S1**	1.45	**S1**	1.08
**H7**	1.60 (1.50)	**H7**	0.74	**H7**	0.84
**H8**	1.59 (2.01)	**H8**	0.74	**H8**	0.84
**H9**	−0.05 (0.08)	**H9**	0.43	**H9**	0.50
< *A*(*CH*_3_) >	1.08 (1.20)		0.64		0.73
< *g* >	2.0120 (2.009)		2.0178		2.0276
